# ERRATUM: Roles of the HOXA10 gene during castrate-resistant prostate cancer progression

**DOI:** 10.1530/ERC-18-0465e

**Published:** 2025-05-15

**Authors:** Zhi Long, Yinan Li, Yu Gan, Dongyu Zhao, Guangyu Wang, Ning Xie, Jessica M Lovnicki, Ladan Fazli, Qi Cao, Kaifu Chen, Xuesen Dong

**Affiliations:** ^1^ Department of Urology, Third Xiangya Hospital, Institute of Prostate Disease, Central South University, Changsha, China; ^2^ Department of Urologic Sciences, Vancouver Prostate Centre, University of British Columbia, Vancouver, British Columbia, Canada; ^3^ Department of Urology, Xiangya Hospital, Central South University, Changsha, China; ^4^ Center for Bioinformatics and Computational Biology, Houston Methodist Research Institute, Houston, Texas, USA; ^5^ Department of Cardiothoracic Surgeries, Weill Cornell Medical College, Cornell University, New York, New York, USA; ^6^ Institute for Academic Medicine, Houston Methodist Hospital, Houston, Texas, USA; ^7^ Department of Urology and Robert H. Lurie Comprehensive Cancer Center, Northwestern University Reinberg School of Medicine, Chicago, Illinois, USA

The authors and journal apologise for an error in the above paper, which appeared in volume **26** part 3, pages 279–292 . The error relates to Fig. 5F given on page 288, in which the AR group CTL and HOXA10 DU145 western blot images were inadvertently duplicated in the AR group CTL and HOXA10 PC3 western blot images. The corrected figure artwork is given in full below.

**Figure 5:**
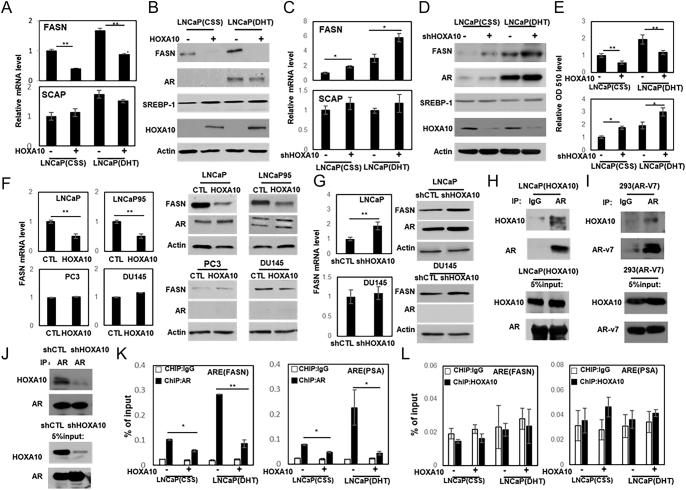
HOXA10 represses FASN expression through the AR. (A and B) LNCaP(CTL) and LNCaP(HOXA10) cells were treated with either vehicle or 10 nM DHT. Real-time PCR and immunoblotting measured FASN and SCAP mRNA levels in (A), and FASN, AR, SREBP1 and HOXA10 protein levels were measured by immunoblotting in (B). (C and D) LNCaP(shCTL) and LNCaP(shHOXA10) cells were treated with either vehicle or 10 nM DHT. Real-time PCR and immunoblotting measured *FASN* and *SCAP* mRNA levels in (C), and FASN, AR, SREBP1 and HOXA10 protein levels were measured by immunoblotting in (D). (E) LNCaP(CTL), LNCaP(HOXA10), LNCaP(shCTL) and LNCaP(shHOXA10) cells were used for Oil Red O staining. Neutral lipid levels were quantified using spectrophotometry at 510 nm wavelength. (F and G) LNCaP, LNCaP95, PC3 and DU145 cells were transfected with control or HOXA10 plasmid (F). HOXA10 expression was depleted in LNCaP and DU145 cells (G). *FASN* mRNA and protein levels were measured by real-time PCR and immunoblotting assays, respectively. (H, I and J) Whole-cell lysates were extracted from LNCaP (H), 293T cells transfected with AR-v7 plasmid (I), or LNCaP cells with HOXA10 knockdown (J) and were used to perform immunoprecipitation assays with either control IgG or an AR antibody. Precipitated proteins were immunoblotted with AR and HOXA10 antibodies. (K and L) LNCaP(CTL) and LNCaP(HOXA10) cells were treated with vehicle or DHT and used to perform ChIP assays with the AR antibody (K) or the HOXA10 antibody (L). Eluted DNA fragments were used as templates for real-time qPCR to measure the enrichment of AR or HOXA10 to androgen response elements in *FASN* and *PSA* promoters. Signals were calculated as percentage of input. All results were derived from three independent experiments performed in triplicate. Data were presented as mean ± S.D. Statistical analyses used one-way ANOVA test followed by Tukey’s test, with *P* < 0.05 as * and *P* < 0.01 as **.

